# The Effects of Ultrasound-Guided Percutaneous Tenotomy on Patients’ Pain and Satisfaction Levels

**DOI:** 10.7759/cureus.57679

**Published:** 2024-04-05

**Authors:** Dylan Parry, Paul Gaschen, Jack Allen, Hillary Beecher, Randy Clark

**Affiliations:** 1 Department of Orthopedic Surgery, Texas Tech University Health Sciences Center, Lubbock, USA; 2 Department of Biology, Utah Tech University, St. George, USA; 3 Department of Orthopedic Surgery, Coral Desert Orthopedics, St. George, USA

**Keywords:** rotator cuff, orthopedics, tendinopathy, tenex, percutaneous tenotomy

## Abstract

Introduction: Tendinopathy is a common pathology with numerous treatment options. Ultrasound-guided percutaneous tenotomy is a newer procedure to treat chronic tendinopathy. It reduces costs and risks compared to other treatments, such as open surgery and platelet-rich plasma (PRP) injections. The goal of percutaneous tenotomy is to induce an acute inflammatory response that recruits clotting and growth factors, induces bleeding, and transforms scar tissue and diseased tendons into a healing state.

Methods: A tenotomy was performed in 57 patients for elbow epicondylitis (13), supraspinatus tendonitis (4), gluteal tendinopathy (34), and patellar tendinopathy (5). The survey was created and sent electronically to all 57 patients, yielding 46 respondents. Each patient was surveyed postoperatively to determine their pain levels on a numeric scale from 1 to 10 prior to and following the procedure. We also asked patients about their satisfaction with the procedure, whether they would recommend it to a friend, and how long it took them to recover completely.

Results: Forty-six of 57 patients responded to the survey. The average healing time was 58 days, and no patients required further surgery. Pain scores significantly improved after tenotomies in the shoulder, elbow, and hip. About 74% of patients were completely satisfied with the procedure, and 80% received enough benefit to recommend it to a friend.

Conclusions: Ultrasonic tenotomy provides significant relief for tendinopathy in the shoulder, elbow, and hip for the majority of patients. The knee pain scores were not significantly reduced, likely due to the small sample size of four patients. Some patients did not experience complete relief and benefited from a PRP injection after tenotomy. Some patients did not benefit, likely due to additional pathology, arthritis, and referred pain. Some limitations to our study include the lack of a control group and each procedure was performed by the same physician, which limits its generalizability. The survey responses were subjective, and the sample size was variable between each body region. More high-quality research is needed to establish the efficacy of tenotomy between different tendons and compare it to other treatment methods.

## Introduction

Tendinopathy is a common pathology with numerous treatment options available. This pathology is characterized by pain, decline in function, and reduced exercise tolerance, and it is commonly found in athletes and adults over 40 years old [1]. The most common overuse tendinopathies include the rotator cuff, common flexor and extensor tendons of the elbow at the medial and lateral epicondyles, patellar tendon, gluteal tendons, and the Achilles tendon [1]. Current conservative treatment options include physical therapy, massage, acupuncture, low-level laser therapy, corticosteroid injections, and nonsteroidal anti-inflammatory drugs. A systematic review in 2011 concluded that these treatments are likely to be beneficial short-term for tendinopathy pain relief, but their long-term efficacy is unestablished [2]. When conservative treatments fail, the ultrasound-guided percutaneous tenotomy via the Tenex Device (Tenex Health, Lake Forest, CA) is available, and its implementation has grown quickly since its first use in 2011 due to the promising results shown in recent years [3].

Percutaneous tenotomy is a newer procedure used to treat chronic tendinopathy. It reduces costs and risks compared to other treatments, such as surgery and platelet-rich plasma (PRP) injections. Open surgical procedures such as tendon debridement introduce more risk, invasiveness, and cost, with patients commonly paying more than $10,000 for the procedure [4]. PRP injections are an alternative treatment option with promising effectiveness and lower costs and risks than open debridement, but they are also more expensive and are frequently not covered by insurance compared to percutaneous tenotomy [4].

The goal of percutaneous tenotomy is to induce an acute inflammatory response in order to recruit clotting and growth factors. By inserting a needle into a chronically diseased tendon, bleeding is induced, the clotting cascade is activated, and growth factors are released. Ultimately, this stimulates scar tissue and transitions diseased tendons into a healing state [5]. This procedure utilizes ultrasound to safely navigate through tissues and is low-risk for bleeding, infection, and other complications associated with any surgery [6]. With the ability to stimulate a healing process in a chronically diseased tendon, percutaneous tenotomy is a promising procedure that can treat chronic tendinopathies, which more conservative treatments have difficulty treating. 

This study aims to further the available literature on percutaneous tenotomy and its effectiveness in reducing pain and improving the quality of life in patients. A limited number of studies have shown that this procedure is low-risk and successful in isolated tendons [7], but few studies compare its effectiveness between different body regions. This paper will help establish the safety and efficacy of percutaneous tenotomy by comparing its use with some of the most common pathologic tendons. In addition, this paper will help to better identify the expected timeframe of patient recovery following the procedure.

## Materials and methods

Procedure

An ultrasound-guided tenotomy was performed in a sterile manner in a single outpatient center by a physician with sports medicine fellowship training. The level of sedation was performed at the request of the patient with the supervision of an anesthesiologist; some patients underwent general anesthesia. Each patient was injected with local anesthesia under ultrasound guidance for the procedure: 8 mL of quarter-percent Marcaine with epinephrine and 3 mL of 1% Lidocaine. A small stab incision was made with an 11-blade scalpel. The device microtip TX2 was guided by the ultrasound to the region of tendinosis through the incision, and the device was activated to irrigate and clear debris with precision via high-frequency oscillation. Any calcifications and tendinopathy were debrided. The TX1 microtip was used for the more superficial treatments at the elbow. The needle was manipulated through the tendon until resolution, as visualized with ultrasound, generally three to five minutes in duration. The incision was closed with steri-strips and bandaged. Patients were advised postoperatively to undergo a gentle range of motion and light activities for two weeks; they could then return to activities as symptoms allow. A post-procedural evaluation was performed in an orthopedic clinic at two weeks, six weeks, and three months.

Survey protocol

Each patient was treated conservatively for at least three months prior to the procedure. Conservative treatment methods performed included physical therapy, anti-inflammatories, and activity modification. Ultrasound-guided percutaneous tenotomy was performed in 57 patients for supraspinatus tendonitis (4), elbow epicondylitis (13), gluteal tendinopathy (34), and patellar tendinopathy (5). These body regions were selected because they are four of the most common pathologic tendons [1], and they are the most common ultrasonic tenotomies performed by the attending physician. The questionnaire was created and sent electronically via email to the 57 patients, yielding 46 respondents who fit the inclusion criteria consisting of male/female patients who were 18 years of age or older, gave appropriate consent, and were diagnosed with chronic tendinopathy by the attending physician. All patients were diagnosed with chronic tendinopathy through magnetic resonance imaging (MRI), clinical assessment, and failure of conservative treatment. Patients with tendon tears were excluded from the procedure and study. In this cohort, information regarding demographics, specific comorbidities, and postoperative complications was documented in each patient’s electronic medical record (EMR). The distributed questionnaire is provided in Appendix A. Each patient was surveyed postoperatively to determine their pain levels on a numeric scale from 1 to 10 prior to and following the procedure. They were additionally asked about their satisfaction with the procedure and if they would recommend it to a friend. Lastly, patients were given a paragraph section in the questionnaire to describe the length of time they needed to make a complete recovery and to provide any additional comments about the procedure. Statistical analysis was performed via a paired t-test for the numeric pain scores before and after the procedure, and P-values were obtained to measure how likely the differences measured were due to chance. Alpha was set at P < 0.05 to determine statistical significance.

## Results

The study included 46 patients, comprising 34 females (73.9%) and 12 males (26.1%). The average body mass index (BMI) of the population was 27.94, categorizing it as overweight. The average age of the patients was 60.65 years. These demographic details are summarized in Table 1.

**Table 1:** Demographics This table has demographic data for the study sample. BMI: body mass index.

Patient demographics	
Female study participants	34 people (73.9%)
Male study participants	12 people (26.1%)
Average age	60.65 years old
Average BMI	27.94 kg/m^2^

**Figure 1:**
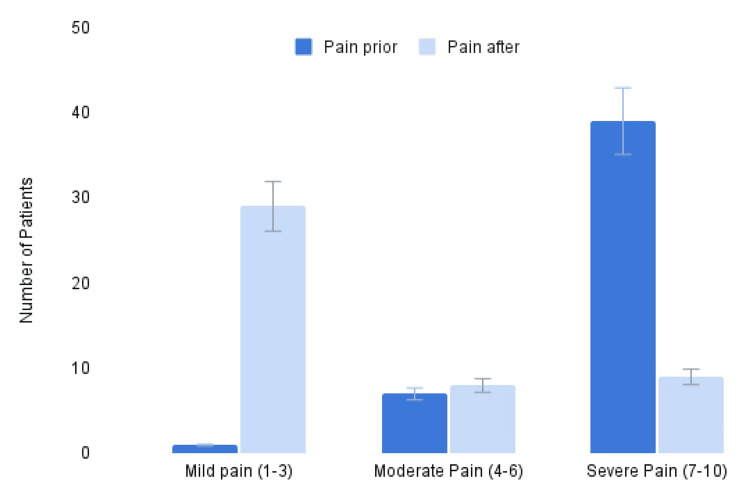
Pain severity Figurative display of the summed values from Table 1, which show pain scores before and after Tenex.

A summary of the interpreted pain scores for each body region is provided in Table 2. The sums are located at the bottom of Table 2, and Figure 1 shows the averages of pain scores before and after the procedure.

**Table 2:** Pain scores Average pain scores before and after Tenex procedure on a scale of 1-10. *Alpha = P < 0.05.

Region	Avg. pain prior	Avg. pain post	P-value
Shoulder (n=3)	8.67	1.33	0.00243*
Elbow (n=10)	7.8	3.3	0.000302*
Gluteal (n=29)	7.93	3.48	7.76E-10*
Knee (n=4)	8.25	4.25	0.0958
Total (n=46)	7.97	3.36	8.52E-16*

In this study, we primarily analyzed the effectiveness of percutaneous tenotomies in reducing patients’ pain levels associated with chronic tendinopathy. We surveyed each of the 57 patients treated with a tenotomy by the same orthopedic surgeon, yielding 46 responses, and examined their subjective pain scores before and after the procedure. When looking at the data from a whole perspective, the method was significantly effective at reducing pain in patients suffering from tendinopathy. When split up by body region, the pain in each region except the patellar tendon (P=0.0958) was significantly reduced.

**Figure 2:**
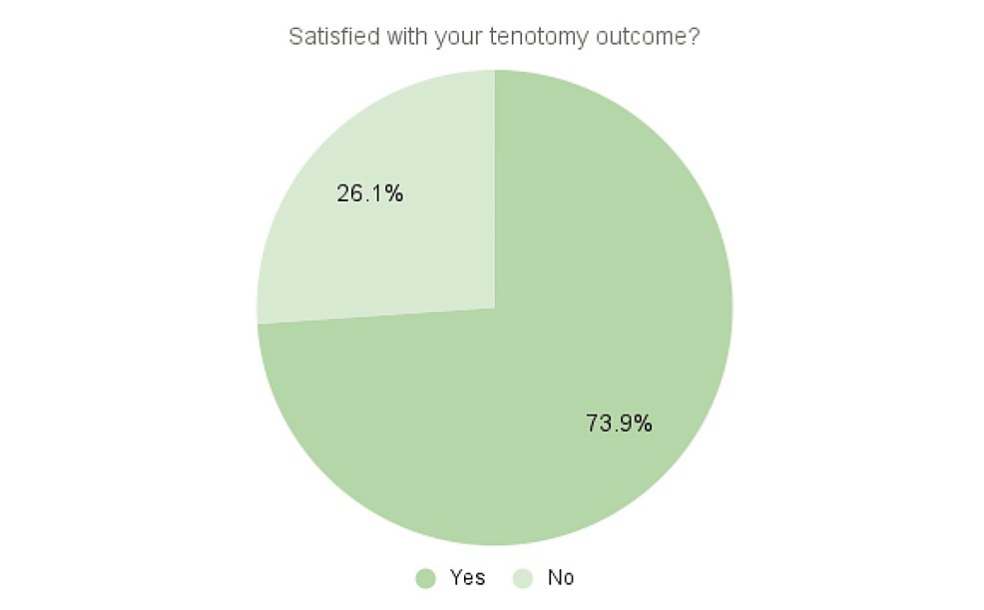
Patient satisfaction This figure contains the averages of the response to whether patient was satisfied with his or her tenotomy outcome.

**Figure 3:**
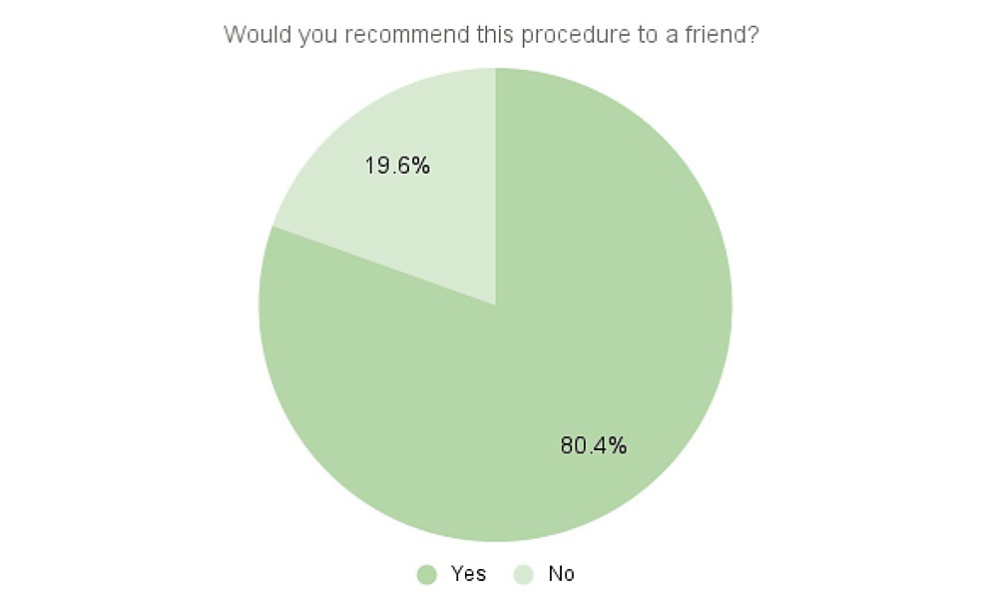
Patient recommendations This figure contains the averages of the response to whether each patient would recommend the procedure to a friend with his or her same condition.

The averages of the “yes or no” questions listed in the Methods section are shown in Figures 2-3.

Thirty-four patients (73.9%) responded yes to whether they were happy with their procedure outcomes. Thirty-seven patients (80.4%) responded that they would recommend the procedure to a friend. There were no complications with bleeding, infection, or hypersensitivity in any of the 57 procedures, and none of the patients required additional surgical intervention for the same pathology. Two of the 46 survey respondents who experienced little to no pain reduction scheduled PRP injections several months after a failed tenotomy.

The subjective survey responses showed that the median time needed to make a significant recovery was 58 days. One patient recovered as quickly as three days, while another took eight months to notice the full pain reduction.

## Discussion

The primary aim of the current research was to supplement the available literature on the efficacy and safety of ultrasonic tenotomies with Tenex Health Devices in multiple body regions. Our data support previous research by showing that this procedure is effective at reducing pain levels in the majority of patients [8-9]. However, this procedure has limitations. The P-values for each tendon region are statistically significant (P<0.05) except for the patellar tendon (P=0.0958). This exception is likely due to the small sample size of only four patients, one of whom experienced no pain reduction. Although one case series showed 100% efficacy and patient satisfaction in percutaneous tenotomy, reducing 8/8 patients’ pain levels with supraspinatus calcific tendinopathy [10], our study showed that some patients had little to no pain reduction, and 26.1% of patients were not satisfied with the outcomes. Our satisfaction results are similar to the findings of a retrospective chart review of 131 patients who underwent ultrasonic tenotomy for elbow tendinopathies [11]. They found significant pain reduction and patient satisfaction in the majority (70%) of patients, but they similarly had patients who did not receive much benefit from the procedure. Perhaps some patients do not benefit due to additional pathology, arthritis, or referred pain. Possible alternative explanations for these results could be that these patients did not have sufficient pain reduction to meet their preoperative expectations, or perhaps their recovery took longer than they initially expected. Regardless, the vast majority of our patients experienced significant pain reduction and were satisfied with the procedure; 80.4% would recommend this treatment to a friend.

In addition to reducing pain, percutaneous tenotomy proved to be a safe and low-risk procedure. Our findings are consistent with previous research showing that this minimally invasive procedure is associated with few risks [12-14]. There were no complications with bleeding, infection, or hypersensitivity in any of the 57 procedures, and none of the patients underwent additional surgery for tendinopathy.

Our findings indicate that ultrasonic tenotomy is likely to heal tendinopathy faster than non-treatment. The subjective survey responses in our study showed that the median time needed to make a significant recovery was 58 days. One patient improved as quickly as three days, while another took eight months to notice pain reduction. One paper indicates that recovery from tendinopathy without surgery takes an average of three to six months [15]. Because more than half of our patients reported significant recovery before three months, our findings show that patients are likely to heal more quickly from an ultrasonic tenotomy compared to the foregoing treatment. 

Some of our patients experienced immediate pain relief, but the majority required longer to notice improvements. While one study indicates steroid injections provide more rapid pain relief from tendinopathy at two weeks post-procedure, their effects are temporary compared to ultrasonic tenotomy long-term [9]. A study by Altahawi et al. showed that pain reduction from ultrasonic tenotomy is most significant between three and six months after the procedure [16]. Because only three patients (6.5% of patients) reported recovery within two weeks, our findings similarly indicate that recovery from the procedure is less likely to be immediate but likely (>50% of patients) to be significant by 58 days.

Some limitations to our study include the lack of a control group and each procedure was performed by the same physician, which limits its generalizability. The survey responses were subjective, which allowed for response bias. The sample size was variable between each body region, which could impact a strict comparison between the different regions. There are limited prospective, randomized trials to date in order to confirm percutaneous tenotomy’s effectiveness between body regions. Such research in the future could help us better understand if certain tendons receive more relief than others, this procedure’s overall effectiveness, and when patients should proceed with it.

## Conclusions

Overall, ultrasound-guided percutaneous tenotomy provides significant pain relief for tendinopathy in the shoulder, elbow, and hip. Most patients experience great pain reduction and are satisfied with the procedure, but some patients do not receive as much benefit as others. With numerous treatment options available, patients and clinicians should discuss whether percutaneous tenotomy is right for them. Patients seeking low-cost and low-risk treatment for advanced tendinopathy after failing conservative treatments should strongly consider the ultrasound-guided percutaneous tenotomy procedure because of its promising ability to reduce pain and improve quality of life. More high-quality research is needed to establish the efficacy of tenotomy between different tendons and compare it to other treatment methods.

## Figures and Tables

**Figure 4:**
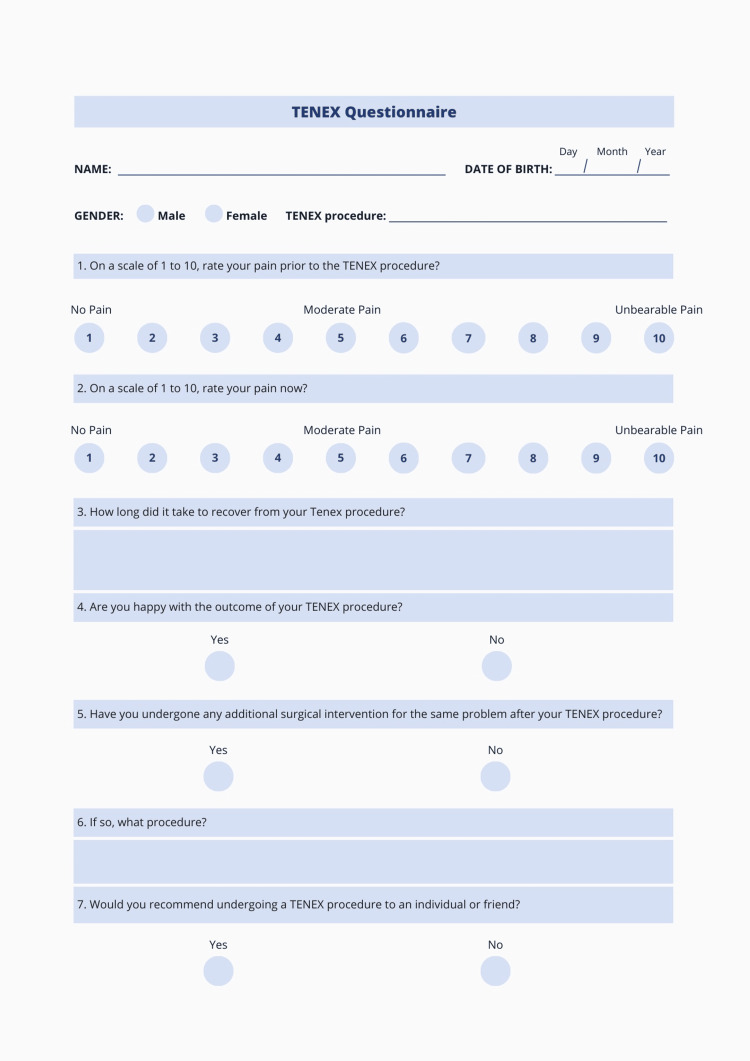
Questionnaire
